# P02.92. The effect of physical activity, obesity, and low vitamin D on all cause mortality in US adults

**DOI:** 10.1186/1472-6882-12-S1-P148

**Published:** 2012-06-12

**Authors:** C Crespo, G Escutia-Dominguez, E Smit, G Brodowicz, S Aden

**Affiliations:** 1 Portland State University, Portland, USA; 2 Oregon State University, Corvallis, USA

## Purpose

To examine the effect of 25-hydroxyvitamin D (serum 25 (OH) D), body mass index and leisure time physical activity on all-cause mortality and determine the association between serum 25 (OH) D and leisure time physical activity among US adults.

## Methods

We used data from 16,285 adults 20 years and older who participated in a home interview and a mobile examination of the Third National Health and Nutrition Examination Survey (1988-1994) that were linked to National Death Index mortality files up to 2006. Physical activity categories included: Inactive (less than 1/wk), Somewhat active (2-4 times/wk), and Active (5+/wk). Body mass index (BMI) included: Underweight (<18.5), Normal weight (18.5-24.9), Overweight (25-29.9), and Obese (30+). Serum 25(OH) D were divided into quartiles, and quartile 1 or low levels included those with values of ≤ 50 nmol/L. Cox proportional hazards were calculated using SAS and SUDAAN softwares to account for sampling weights of NHANES.

## Results

In the final model we adjusted for age, sex, race/ethnicity, education, smoking status, region of the country, and presence of chronic diseases plus the three variables of interest: physical activity, body mass index, and serum 25(OH)D. Low serum vitamin D was a significant predictor of all cause mortality independent of physical activity and obesity status; and while being physically active 5 or more times a week had a protective effect (RR=0.65, 95 CI=0.58,0.74), obesity was not significantly related (1.06, 95 CI=0.96-1.18) with all cause mortality.

## Conclusion

We found that low Vitamin D and physical inactivity were independent risk factors for all-cause mortality whereas obesity was not an independent risk factor when adjusting for physical activity and serum Vitamin D levels. More research is needed to understand the role of adiposity in serum 25(OH)D metabolism.

